# A first-principles study of electronic structure and photocatalytic performance of GaN–MX_2_ (M = Mo, W; X= S, Se) van der Waals heterostructures

**DOI:** 10.1039/d0ra04082g

**Published:** 2020-06-29

**Authors:** Fawad Khan, M. Idrees, C. Nguyen, Iftikhar Ahmad, Bin Amin

**Affiliations:** Department of Physics, University of Malakand Chakdara 18800 Pakistan; Department of Physics, Hazara University Mansehra 21300 Pakistan; Institute of Research and Development, Duy Tan University Da Nang 550000 Vietnam nguyenvanchuong2@duytan.edu.vn; Department of Physics, Abbottabad University of Science and Technology Abbottabad 22010 Pakistan binukhn@gmail.com

## Abstract

Modeling novel van der Waals (vdW) heterostructures is an emerging field to achieve materials with exciting properties for various devices. In this paper, we report a theoretical investigation of GaN–MX_2_ (M = Mo, W; X= S, Se) van der Waals heterostructures by hybrid density functional theory calculations. Our results predicted that GaN–MoS_2_, GaN–MoSe_2_, GaN–WS_2_ and GaN–WSe_2_ van der Waals heterostructures are energetically stable. Furthermore, we find that GaN–MoS_2_, GaN–MoSe_2_ and GaN–WSe_2_ are direct semiconductors, whereas GaN–WS_2_ is an indirect band gap semiconductor. Type-II band alignment is observed through PBE, PBE + SOC and HSE calculations in all heterostructures, except GaN–WSe_2_ having type-I. The photocatalytic behavior of these systems, based on Bader charge analysis, work function and valence and conduction band edge potentials, shows that these heterostructures are energetically favorable for water splitting.

## Abstract 2

Modeling novel van der Waals (vdW) heterostructures is an emerging field to achieve materials with exciting properties for various devices.

## Graphical abstract

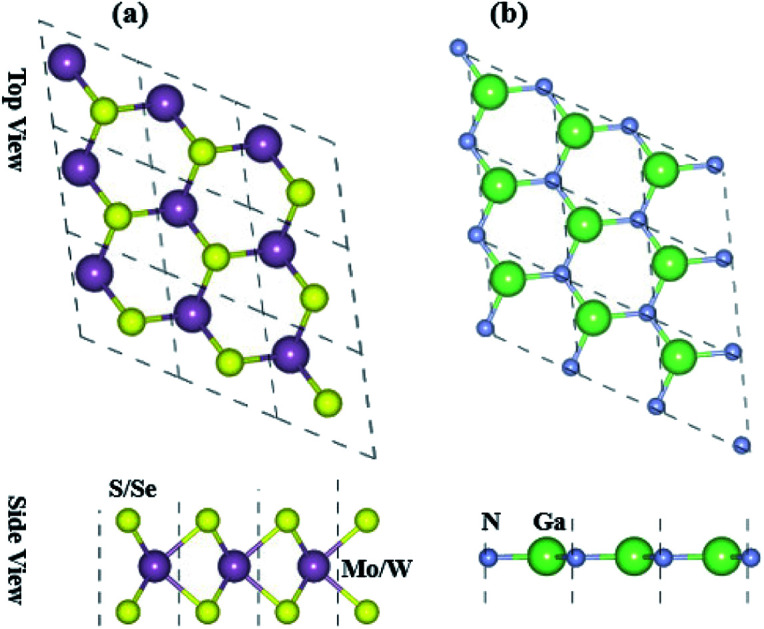

## 1 Introduction

Developing material with desired characteristics for specific applications is the most challenging aspect of modern science and technology. Graphene is favored for its extraordinary properties, such as high Young's modulus and high electrical conductivity.^1,2^ Graphene is a zero band gap material,^2^ although it ensures excellent electrical properties. The lack of band gap prevents its use in low-power electronic switching logic circuits.^3,4^ After graphene, the research fields are dominated by layered TMDCs like disulfide, diselenide, ditelluride and fluoride.^5,6^ The TMDC family has more than 40 different combinations of multi-layer elements, such as black phosphorous^7^ and five layers of selenium.^8^

MoS_2_ is a prominent TMDC with a layered structure, capable of converting from an indirect band gap of 1.2 eV to a direct band gap of 1.9 eV,^9,10^ when reduced from bulk to single layer. Monolayer MoS_2_ has mobility of 200 cm^2^ V^−1^ s^−1^ and high on/off ratio at room temperature,^11^ which makes it suitable material for many applications in optoelectronics.^12^ Like MoS_2_, when the layers of MoSe_2_ is reduced to several are single layer, the nature of band gap (1.1 eV) changes to a direct (1.55 eV).^13^ Monolayer MoSe_2_ is a semiconductor having high efficiency of photoconversion, which makes it suitable for high-efficiency electronic devices. Interlayer spacing in MoSe_2_ is 0.646 nm, which is comparatively wider than MoS_2_ (0.615 nm) and graphite (0.335 nm).^14,15^ The tuning of band gap in MoSe_2_ is possible by applying mechanical strain or external electric field. Therefore, MoSe_2_ is considered as an ideal material in optoelectronic devices such as light emitting diodes^16^ and field effect transistors.^17^ The large number of active sites, stability and low cost make MoSe_2_ as an efficient catalyst for hydrogen evaluation reaction.^18^

The large size of tungsten has attracted the attention of researches on tungsten chalcogenides 2D structure. The natural abundance of tungsten is similar to Mo but is heavier element than Mo. Furthermore, Mo is highly consumed in industries while it has lower consumption, which makes tungsten more advantageous in industrial applications. WSe_2_ is used in various applications like photodetectors^19–21^ and field effect transistors.^22–26^ Like other TMDCs, in WSe_2_ the W element is sandwiched between two layers of Se atoms. The properties of MoS_2_, MoSe_2_, WS_2_ and WSe_2_ are similar to each other and hence it is appropriate to study these TMDCs altogether.^27–29^

More recently, a novel 2D graphene-like gallium nitride (GaN) has been synthesized through migration-enhanced encapsulated growth.^30^ 2D GaN is known to be an indirect band gap semiconductor.^31^ Moreover, the electronic properties of GaN monolayer are very sensitive to other conditions, such as electric field,^32^ surface functionalization^33^ and constructing heterostructures.^34,35^ These investigations demonstrate that 2D GaN monolayer can be considered as a promising candidate for high-performance optoelectronic and nanoelectronic devices.^36–38^ GaN is also attempted for photocatalytic water splitting due to its high thermal conductivity and good chemical and thermal stability.^39^

The energy crisis and environment problem can be easily resolved if sufficient and clean energy could be achieved through photocatalysis. After the catalytic water splitting on TiO_2_ electrodes, huge number of photocatalyst are developed.^40^ When a photocatalytic material is exposed to sunlight, electron–hole pairs are produced. In photocatalysis the material acts as a catalyst for solar energy conversion and H_2_ is produced from water, when it is exposed to light^41,42^ that can be utilized as a source of green energy. A low photocatalytic activity in semiconducting material is due to the easily recombination of excited and unstable electrons and holes.^43^ To increase the photocatalytic activity it is important to design materials with diverse properties like crystal structure,^44^ particle size^45^ and crystallinity.^46^ The separation and migration of charges in these materials are efficient and light absorbing range is broader. For an efficient photocatalyst several requirements must be critically satisfied, such as the band gap of the semiconductor must be suitable and with respect to water redox potential such that the conduction band minimum and the valance band maximum must have reasonable positions.^47,48^ In layered material the optical absorption can be tuned by adjusting its band gap and thus can be considered advantageous as a photocatalyst in comparison to other conventional materials, while the recombination of photogenerated holes and electrons are much decreased due to high carrier mobility and ultrathin nature. The performance of photocatalysis in layered materials is also increased due to its large surface area with potentially reactive sites.^49^

Modeling novel van der Waals (vdW) heterostructure is an emerging field to achieve materials with exciting properties for various devices. In a vdW heterostructure, the layers are assembled in a precisely controlled sequence,^50,51^ which provides a platform for designing novel materials with new phenomena in nanoelectronics. In these heterostructures, the interlayer interaction is vdW, which offers a wide range of materials based on the lattice matching of the individual layers. So far, a large number of vertically stacked vdW heterostructures are experimentally and theoretically investigated, and they are found with good properties for electronic and optoelectronic applications.^52^ Heterostructure photocatalysts are promising materials with better photocatalytic properties than that of the individual layers.^53^ In a formed heterostructure, the band gap width and positions are effectively tuned to reach the requirement of a photocatalyst. In a type-II heterostructure, having reasonable values, the hydrogen and oxygen are generated at the opposite side of the material, and hence the holes and electrons can be effectively separated, which leads to an improvement in the water splitting efficiency.^54^

Therefore, in this work, considering all the superior properties of both monolayers GaN and MX_2_ (M = Mo, W; X = S, Se), we construct the vdW heterostructures based on GaN and MX_2_ monolayers and investigate their electronic properties and photocatalytic response using first principles calculations. Our results indicate that GaN–MoS_2_, GaN–MoSe_2_, GaN–WS_2_ and GaN–WSe_2_ van der Waals heterostructures are energetically stable. Furthermore, the GaN–MoS_2_, GaN–MoSe_2_, GaN–WS_2_ heterostructures form type-II band alignment, in which the oxidation and reduction reaction will be induced at different layers. The photocatalytic behavior of these heterostructures that based on Bader charge analysis, work function and valence and conduction band edge potentials, show that these heterostructures are energetically favorable for water splitting.

## 2 Computational details

First-principles calculations based on density functional theory (DFT)^55^ are performed with Vienna *ab initio* simulation package (VASP) using the projector augmented wave (PAW) method.^56^ The Perdew–Burke–Ernzerhof (PBE) form of generalized gradient approximation (GGA)^57^ and the Heyd–Scuseria–Ernzerhof hybrid functional (HSE06)^58^ are used to describe the electron exchange and correlation energy. The DFT long-range dispersion correction (DFT-D2) method proposed by Grimme,^59^ is adopted for vdW correction to the PBE functional. Spin orbit coupling (SOC) effect is significant in TMDCs and Janus monolayers hence; SOC is also taken into account in our calculations. The SOC is incorporated by a second variational method,^60^ which uses scalar-relativistic basis, based on the reduction of original basis. In variation method, the scalar relativistic part of Hamiltonian is diagonlized in scalar relativistic basis and the calculated eigen functions are then used to construct the full Hamiltonian matrix including SOC. The kinetic energy cutoff of the plane wave is set to 500 eV. All the geometric relaxation and self consistent iteration are finished until the force on each atom and energy difference between electronic steps are converged to 0.001 eV Å^−1^ and 10^−5^ eV, respectively. The *k*-point mesh of the Brillouin zone integration is sampled by a 6 × 6 × 1 for geometric relaxation and 12 × 12 × 1 centered *k*-mesh for the optimized structures to achieve high accuracy. The vacuum along *z*-direction is set to 25 Å to avoid artificial interaction between the adjacent slabs.

## 3 Result and discussion

**Fig. 1:**
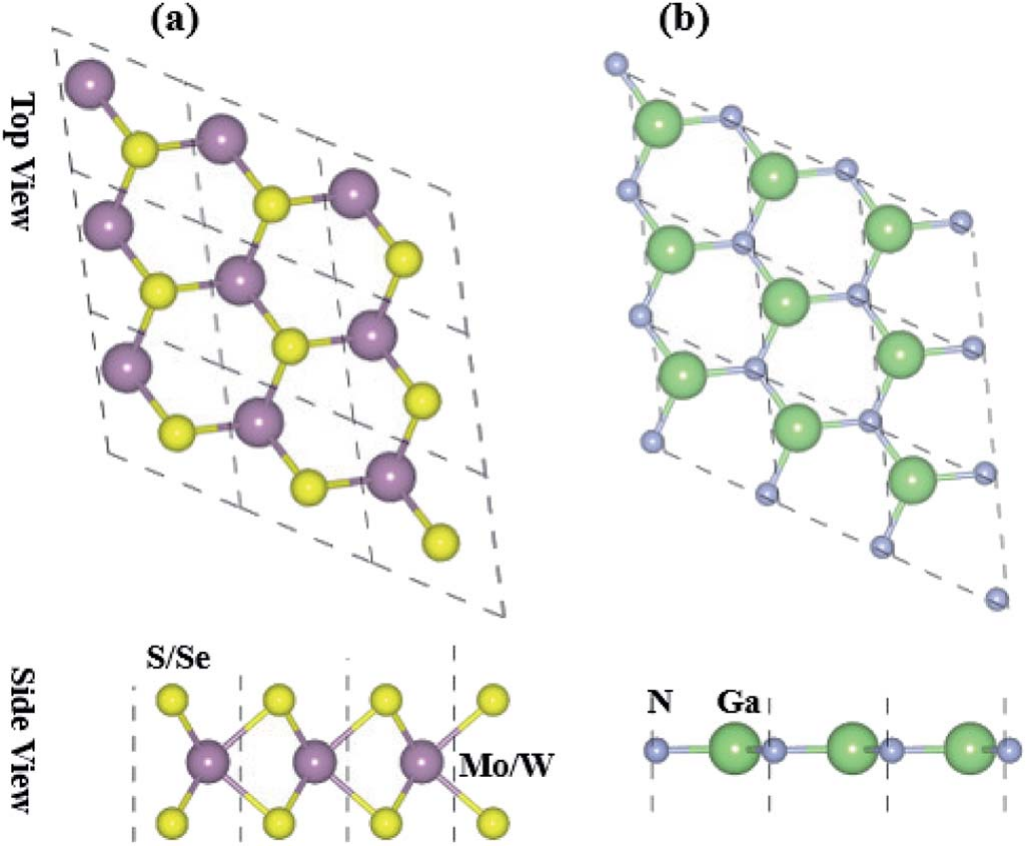
Top and side view of the free standing (a) MX_2_ (M = Mo, W; X = S, Se) and (b) GaN monolayers.

Optimized structures of freestanding TMDCs and GaN monolayers are shown in Fig. 1. The calculated lattice constants of GaN, MoS_2_, WS_2_, MoSe_2_ and WSe_2_ are 3.25 Å, 3.16 Å, 3.29 Å, 3.17 Å and 3.29 Å, respectively, which show the small lattice mismatch of less than 3%. It is quite difficult to control the orientation of monolayers in mechanical exfoliation and/or subsequent staking in fabrication of 2D vdW heterostructures. Layer stacking can effectively modulate the electronic structure in the formation of vdW heterostructures.^61^ Therefore, using the optimized lattice constant of GaN and TMDCs monolayers, four possible stacking configurations in the form of GaN–MX_2_ (M = Mo, W; X = S, Se) vdW heterostructures are investigated. In stacking (I) the Ga atom placed on the top of Mo/W atom, while the N atom on the top of S/Se atom. In stacking (II) the Ga atom placed on the top of S/Se atom while the N atom on the top of M/W atom. In stacking (III) the Ga atom placed on the top of M/W atom while the N atom is in the hexagonal site. In stacking (IV) the N atom placed on the top of M/W atom while the Ga atom is in the hexagonal site. We check the structural stability of these heterostructures by calculating the binding energy, interlayer distance and thermal stability. Binding energy is the difference in the total energy of the heterostructures and their parent monolayers as follows:

1 *E*_b_ = *E*_GaN–MX_2__ − *E*_GaN_ − *E*_MX_2__

**Fig. 2:**
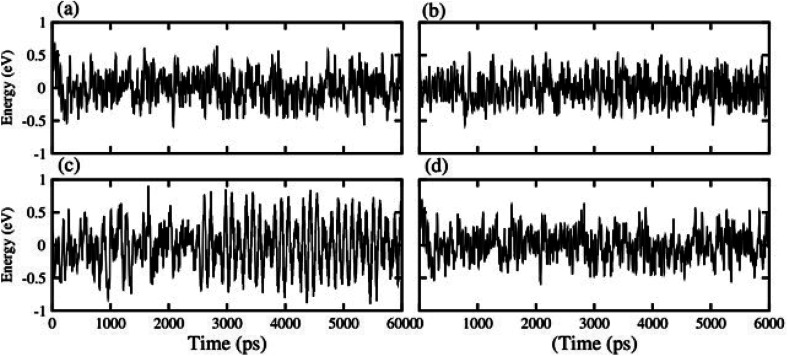
Thermal stabilities of (a) GaN–MoS_2_ (b) GaN–MoSe_2_, (c) GaN–WS_2_ and (d) GaN–WSe_2_ van der Waals heterostructures.

Here, *E*_GaN–MX_2__, *E*_GaN_ and *E*_MX_2__, respectively, are the total energies of GaN–MX_2_ heterostructure, the constituent GaN and MX_2_ monolayers. The binding energy and interlayer distance of GaN–MX_2_ heterostructures for different stacking configurations are listed in Table 1. One can find that the interlayer distances of GaN–MX_2_ heterostructures for all stacking configurations are in the range of (3.21 ÷ 3.33) Å. These values are comparable with typical vdW systems,^62^ confirming that all these heterostructures are formed by vdW forces. Smaller binding energy and shorter interlayer distance of the GaN–MX_2_ heterostructure demonstrate that the stacking (II) is the most energetically favorable stacking configuration. The AIMD simulations of the GaN–MX_2_ heterostructures for the most energetically favorable stacking configurations are also performed and displayed in Fig. 2 to confirm their thermally stable at room temperature. One can see that the fluctuations in total energy of the GaN–MX_2_ heterostructures before and after 6 ps are quite small, demonstrating that the GaN–MX_2_ heterostructures are thermal stability at room temperature.

**Table 1:** Binding energies (*E*_I_, *E*_II_, *E*_III_, *E*_IV_ in eV), interlayer distance (*D* in Å), lattice constant (*a* in Å), band gap (*E*_g_ in eV), work function (*Φ*), conduction and valence band edges (*E*_CBM_, *E*_VBM_ in eV) of GaN–TMDCs heterostructure

Heterostructures	GaN–MoS_2_	GaN–MoSe_2_	GaN–WS_2_	GaN–WSe_2_
*E*_I_/*D*_I_	−0.2096/3.28	−0.2775/3.27	−0.2902/3.26	−0.3291/3.26
*E*_II_/*D*_II_	−0.2118/3.24	−0.2776/3.21	−0.2905/3.22	−0.3292/3.24
*E*_III_/*D*_III_	−0.2116/3.29	−0.2413/3.30	−0.2653/3.33	−0.2871/2.28
*E*_IV_/*D*_IV_	−0.2049/3.27	−0.2274/3.31	−0.2563/3.25	−0.2695/3.29
*a*	3.21	3.28	3.23	3.29
*E*_g_ (PBE)	1.415	1.425	1.563	1.659
*E*_g_ (PBE + SOC)	1.397	1.242	1.354	1.334
*E*_g_ (HSE)	2.139	1.584	2.10	2.27
*Φ*	1.383	1.527	1.497	1.009
*E*_VBM_	1.317	1.120	1.510	1.445
*E*_CBM_	−0.098	−0.121	−0.053	−0.213

**Fig. 3:**
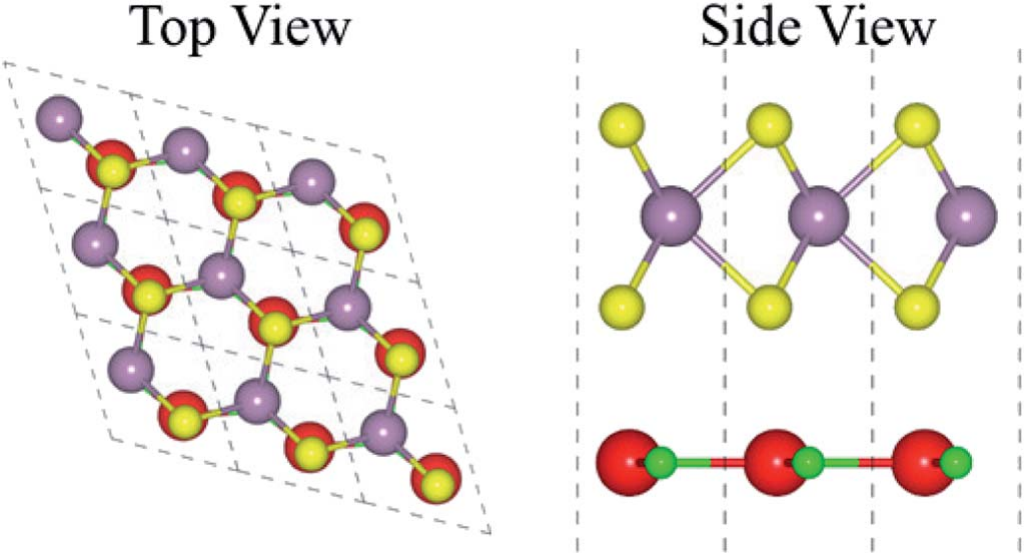
Top and side view of the stable staking of GaN–MX_2_ (M = Mo, W; X = S, Se), where red (green) represent the Ga (N) atoms while yellow (gray) represents the Mo/W (S/Se) atoms, respectively.

**Fig. 4:**
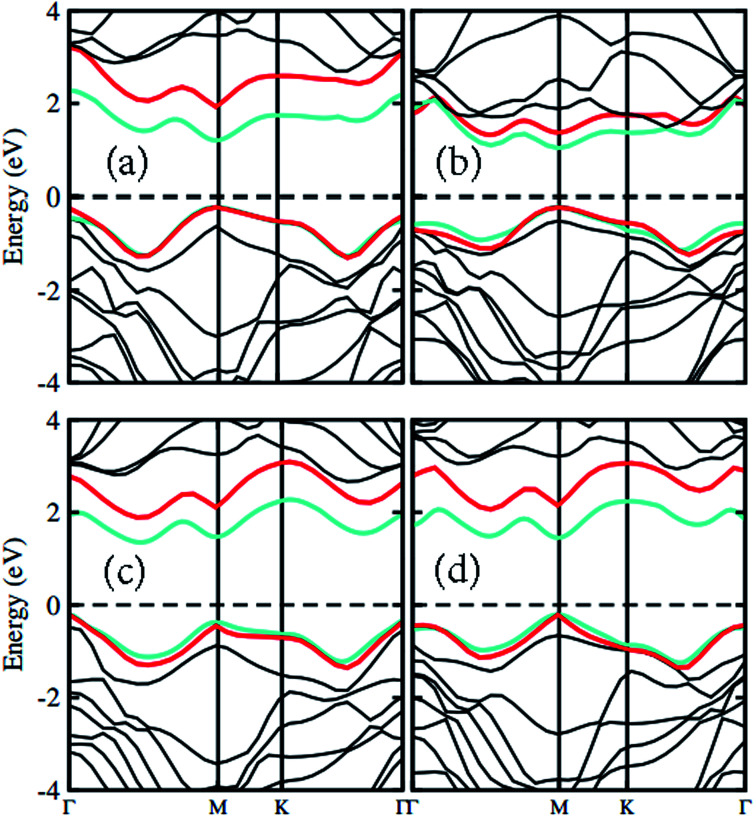
Band structures of (a) GaN–MoS_2_, (b) GaN–MoSe_2_, (c) GaN–WS_2_ and (d) GaN–WSe_2_ respectively. Red and light green represents the PBE and HSE06 calculations, respectively.

**Fig. 5:**
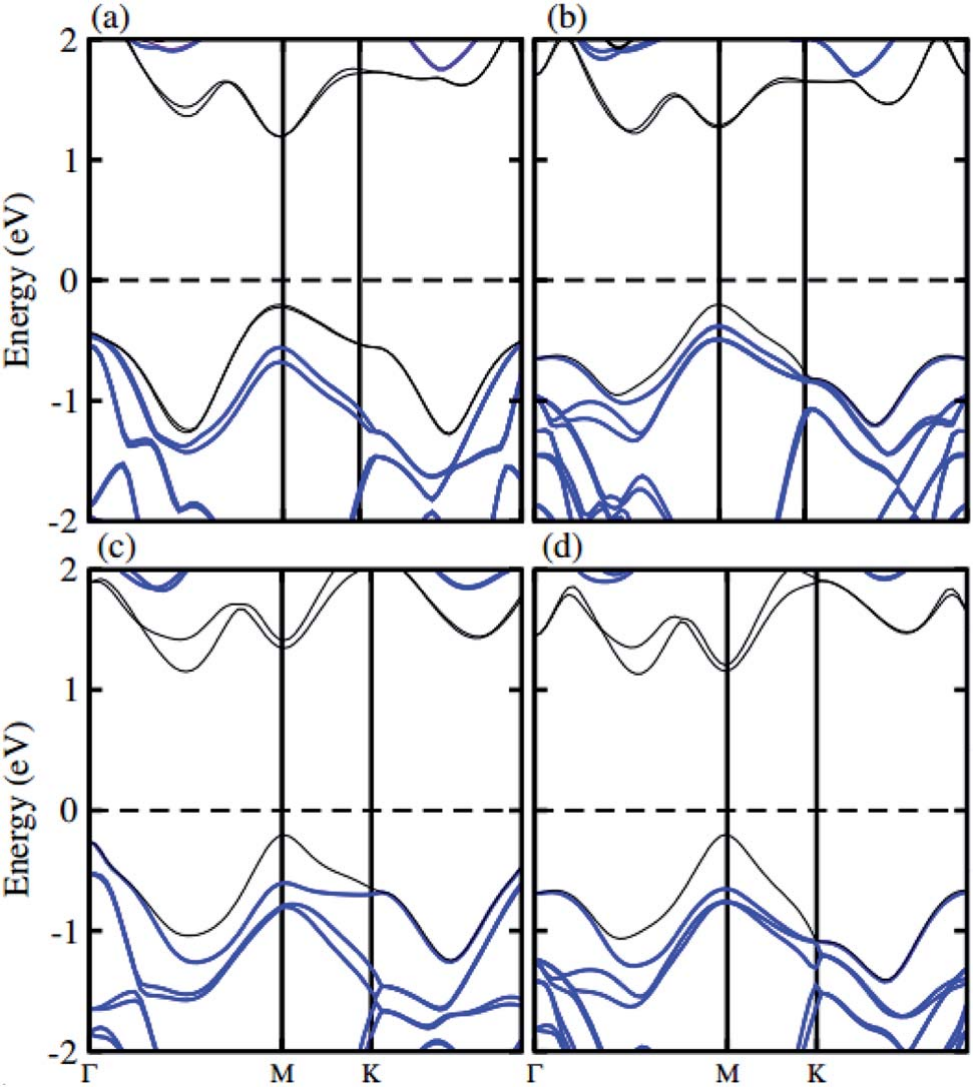
Band structure of (a) GaN–MoS_2_, (b) GaN–MoSe_2_, (c) GaN–WS_2_ and (d) GaN–WSe_2_ respectively with SOC effect.

The atomic structure of the GaN–MX_2_ heterostructures for the most energetically favorable stacking configuration (stacking II) is depicted in Fig. 3. The electronic band structures of GaN–MX_2_ (M = Mo, W; X= S, Se) heterostructures in stacking II calculated by PBE and HSE06 are displayed in Fig. 4 as well as their band structures given by PBE + SOC in Fig. 5. Moreover, the band gap values of GaN–MX_2_ heterostructure for different methods are listed in Table 1. For PBE GaN–MoS_2_Fig. 4(a) vdW heterostructures shows direct band gap semiconductor nature with CBM and VBM at the M point of Brillion zone. It is clear that PBE method underestimates the band gap values, therefore, we have also used the HSE06 method for obtaining more accurate band gap. Obviously, for HSE06 method in Fig. 4(a), the GaN–MoS_2_ vdW heterostructures also shows semiconductor with direct band gap of 2.139 eV, which is still smaller than that of both monolayers, indicating that the formation of heterostructure results in a reduction of the band gap values. Interestingly, these band gap values of heterostructures are still larger than the minimum required band gap for the photocatalytic reaction (1.23 eV), showing the potential applications of these vdW heterostructures as a visible light photocatalyst. Inducing spin–orbit coupling (SOC) effects in GaN–MoS_2_ heterostructure split CBM and VBM, hence reduce the band gap values, while the nature of band gap remains unchanged (see Fig. 5(a)). In case of GaN–MoSe_2_, GaN–WS_2_ and GaN–WSe_2_ vdW heterostructures the GaN–MoSe_2_ and GaN–WSe_2_ shows direct band nature while GaN–WS_2_ shows indirect band nature with the band gap value given in Table 1 for PBE/PBE + SOC/HSE06 method. Among these methods, HSE06 method gives more accurate band gaps of GaN, MX_2_ monolayers and their heterostructures and thus, it can be concluded that the HSE06 method provides the band gap values that are more consistent with experimental measurements.

**Fig. 6:**
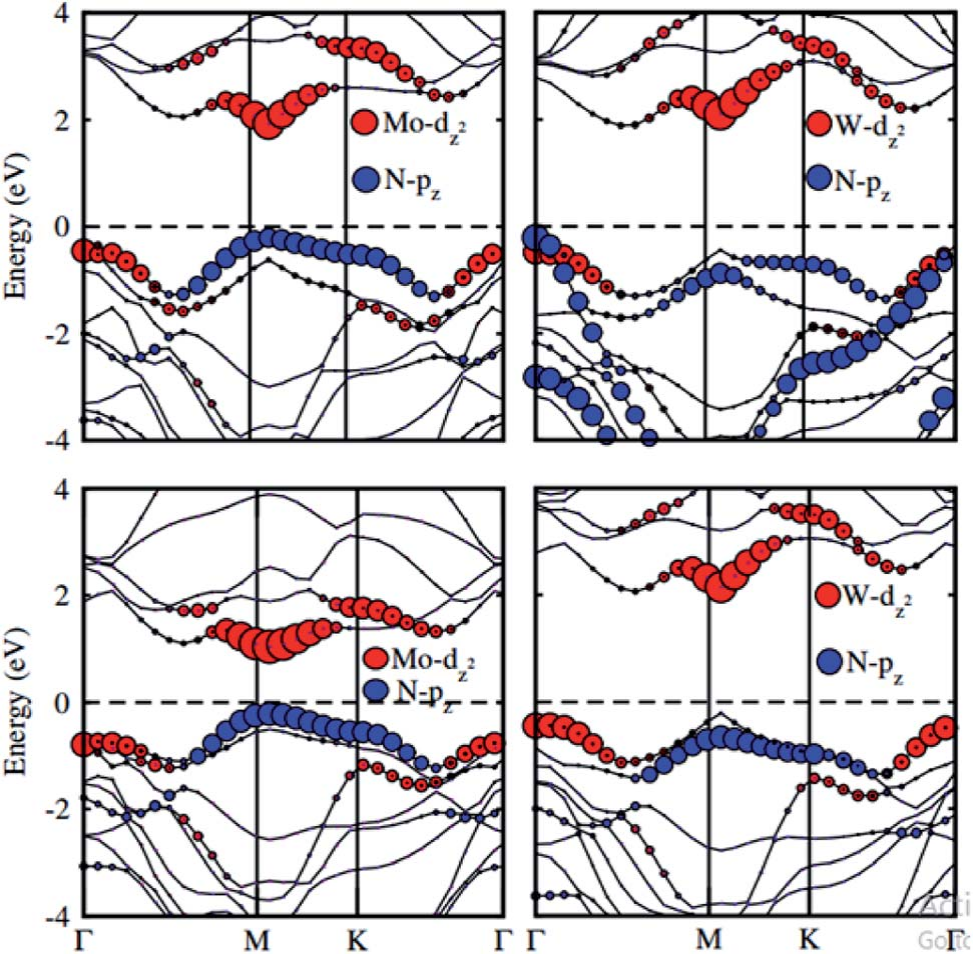
Weighted band structure of GaN–MX_2_ (M = Mo, W; X = S, Se).

To check the contributions of substates in the VBM and CBM we further calculate the weighted band structures of these vdW heterostructures, as depicted in Fig. 6. One can observe that the GaN–MoS_2_, GaN–WS_2_ and GaN–MoSe_2_ heterostructures form the type-II band alignment. The VBM of the GaN–MoS_2_, GaN–WS_2_ and GaN–MoSe_2_ heterostructures comes from N-p_*z*_ orbitals of the GaN layer, whereas their CBM is contributed by the MX_2_ layer. The type-II band alignment will spontaneously separate the free electrons and holes, resulting high efficiency in optoelectronic and solar energy conversion. On the contrary, the GaN–WSe_2_ heterostructure possesses the type-I band alignment. Both the VBM and CBM of GaN–WSe_2_ heterostructure come from the W-d_*z*^2^_ orbitals of the WSe_2_ layer, confirming the formation of the type-I band alignment.

In order to gain a deep insight into the origins of the enhanced photocatalytic performance, we calculate charge density difference (Δ*ρ*) which is defined as:

2 Δ*ρ* = *ρ*_GaN–MX_2__ − *ρ*_GaN_ − *ρ*_MX_2__

**Fig. 7:**
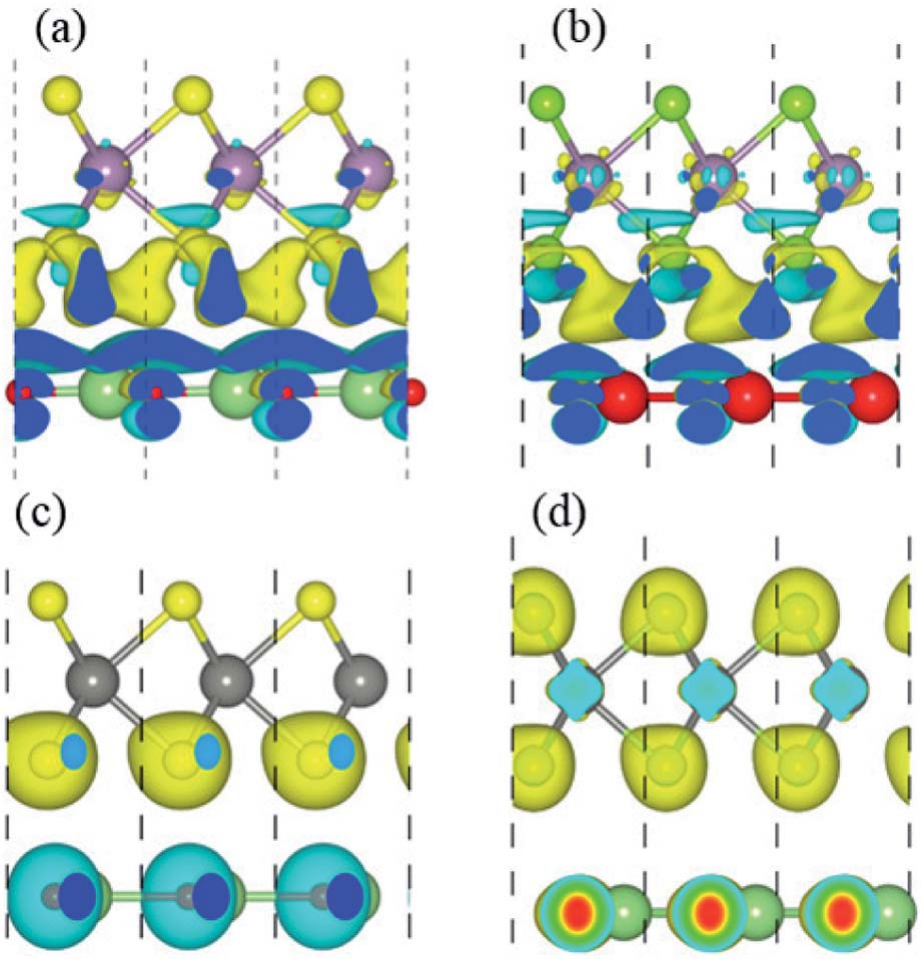
Charge differences of (a) GaN–MoS_2_, (b) GaN–MoSe_2_, (c) GaN–WS_2_ and (d) GaN–WSe_2_ respectively. The iso-value chosen to plot the iso-surface is 0.001 eV Å^−3^.

Here, *ρ*_GaN–MX_2__, *ρ*_GaN_ and *ρ*_MX_2__, respectively, are the charge densities of GaN–MX_2_ heterostructure, isolated GaN and MX_2_ monolayers. The charge density difference in the GaN–MX_2_ heterostructures are depicted in Fig. 7, where the blue dotted represent the electrons gain and the yellow for electrons lose. From Table 2, it is clear that for all heterostructures the charges are transfer from the GaN monolayer to the MX_2_ layers. It also demonstrates that in these heterostructures, the GaN become n-type doped semiconductor while the MX_2_ layer become p-type doped semiconductor. Hence these results also confirm type-II band alignment, which slows down charge recombination and is highly desirable for light harvesting applications.

**Table 2:** Bader charge distribution in the GaN–TMDCs vdW heterostructures

Heterostructures	GaN–MoS_2_	GaN–MoSe_2_	GaN–WS_2_	GaN–WSe_2_
Mo	0.0069	0.006	—	—
W	—	—	−1.0312	0.0068
Si	−0.0007	—	0.1183	—
Si	0.023	—	0.9523	—
Sei	—	−0.0035	—	−0.0019
Seii	—	0.013	—	0.0117
Ga	0	0	0	0
N	−0.0292	−0.0155	−0.0395	−0.0166

The surface conditions of every material can easily affect the work function, which results from altering the surface electric field induced by the distribution of electrons at the interface. The work function is the amount of energy required to remove an electron from the Fermi level surface of a solid vacuum at an absolute zero. The work function along the *z* direction is calculated by aligning the Fermi energy level with reference to the vacuum energy level:^63^*Φ* = *E*_vacuum_ − *E*_Fermi_, where *E*_vacuum_ and *E*_Fermi_ are the energy of an electron at the stationary point in the vacuum with respect to the surface and Fermi level, respectively. The work function of the heterostructure is presented in Table 1. The work functions of the GaN–MX_2_ heterostructures are lower than those of the constituent monolayers because of the efficient interfacial formation and charge transfer at the interface.

**Fig. 8:**
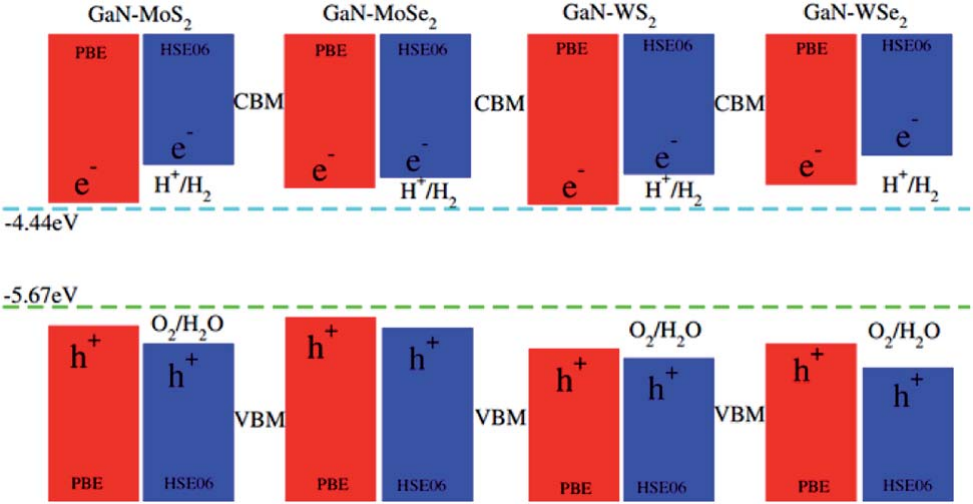
Valence and conduction band edge potentials of GaN–MX_2_ (M = Mo, W; X = S, Se), red for PBE while the blue represents the HSE06 method.

To check the performance of GaN–TMDCs heterostructures we have calculated the photocatalytic response of these heterostructure at pH = 0 using Mulliken electronegativity.^64,65^ The equation for Mulliken electronegativity for VBM and CBM is given by: *E*_VBM_ = *χ* − *E*_elec_ + 0.5*E*_*g*_ and *E*_CBM_ = *E*_VBM_ − *E*_g_. In these equations the *χ* represents the geometric mean of Mulliken electronegativities of the constituent atoms, *E*_elec_ is constant value of 4.5 eV and *E*_g_ represents the band gap values. The band alignment of these heterostructures with respect to the oxidation–reduction reaction for water splitting at pH = 0 for PBE and HSE06 methods is depicted in Fig. 8. The water reduction potential and the oxidation potential with respect to the vacuum level are −4.44 eV and −5.67 eV, respectively. From the Fig. 8 it is clear that for PBE calculation (red lines) for all heterostructures are energetically suitable positions of the band edges which are just outside of the reduction and oxidation potentials shows good response for water splitting at pH = 0. As GaN–TMDCs shows good response for water splitting at pH = 0 at PBE level, so for HSE06 the band gap of these heterostructures further increasing and show more energetic response for water splitting at pH = 0. It indicates that in all these GaN–MX_2_ heterostructures, both the reduction potential *E*_H^+^/H_2__ and the oxidation potential *E*_O_2_/H_2_O_ are lied between their VBM and the CBM. It means that for all the GaN–MX_2_ heterostructures, the VBM locates more positive than the water oxidation potential, whereas the CBM is more negative than the hydrogen reduction potential. These findings make these heterostructures promising candidate for photocatalytic water splitting. In addition, owing to the formation of the type-II band alignment, the redox reactions of the GaN/MX_2_ heterostructures occur in different layers, boosting for the separation of photogenerated charges. Therefore, we can conclude that the GaN–TMDCs heterostructures are efficient photocatalysts for conversion of solar light into hydrogen, which is an attractive technique for the production of clean and renewable energy device applications.

## 4 Conclusion

In summary, we have investigated systematically GaN–TMDCs heterostructures in detail, such as the electronic properties, charge density difference, work function, band alignment and photocatalytic properties using first-principles calculations. The thermal stability and binding energy studies confirm stability of GaN–MoS_2_, GaN–MoSe_2_, GaN–WS_2_ and GaN–WSe_2_ heterostructures. It is found that GaN–MoS_2_, GaN–MoSe_2_ and GaN–WSe_2_ heterostructures are direct band gap materials while GaN–WS_2_ is indirect band gap semiconductor. Type-I band alignment is confirmed in GaN–WSe_2_ and type-II in GaN–MoS_2_, GaN–MoSe_2_ and GaN–WS_2_. Interlayer charge transfer shows those electrons are transferred from GaN to TMDCs monolayers. The photocatalytic behavior of these systems reveals that these heterostructures are suitable for water splitting at zero pH.

## Conflicts of interest

There are no conflicts to declare.

## Supplementary Material
